# Association of changes in obesity and abdominal obesity status with early-onset colorectal cancer risk: a nationwide population-based cohort study

**DOI:** 10.3389/fmed.2023.1208489

**Published:** 2023-06-21

**Authors:** Ji Hyun Song, Ji Yeon Seo, Eun Hyo Jin, Goh Eun Chung, Young Sun Kim, Jung Ho Bae, Sunmie Kim, Kyung-Do Han, Sun Young Yang

**Affiliations:** ^1^Department of Internal Medicine and Healthcare Research Institute, Healthcare System Gangnam Center, Seoul National University Hospital, Seoul, Republic of Korea; ^2^Department of Obstetrics and Gynecology, Healthcare Research Institute, Healthcare System Gangnam Center, Seoul National University Hospital, Seoul, Republic of Korea; ^3^Department of Statistics and Actuarial Science, Soongsil University, Seoul, Republic of Korea

**Keywords:** early onset colorectal cancer, obesity, abdominal obesity, obesity change, cohort study

## Abstract

**Background and aims:**

The incidence of early-onset colorectal cancer (EO-CRC, diagnosed before 50 years of age) has increased in recent decades. The aim of this study was to investigate the association between changes in obesity status and EO-CRC risk.

**Methods:**

From a nationwide population-based cohort, individuals <50 years old who participated in the national health checkup program in both 2009 and 2011 were included. Obesity was defined as a body mass index ≥25 kg/m^2^. Abdominal obesity was defined as a waist circumference ≥ 90 cm in men and ≥ 85 cm in women. Participants were classified into 4 groups according to the change in obesity (normal/normal, normal/obese, obese/normal, persistent obese) and abdominal obesity (normal/normal, normal/abdominal obesity, abdominal obesity/normal, persistent abdominal obesity) status. Participants were followed up until 2019 and censored when they became 50 years old.

**Results:**

Among 3,340,635 participants, 7,492 patients were diagnosed with EO-CRC during 7.1 years of follow-up. The risk of EO-CRC was higher in the persistent obesity and persistent abdominal obesity groups than in the normal/normal groups (hazard ratio (HR) [95% confidence interval (CI)] = 1.09 [1.03–1.16] and 1.18 [1.09–1.29], respectively). Participants with both persistent obesity and abdominal obesity had a higher EO-CRC risk than those in the normal/normal groups for both [HR (95% CI) = 1.19 (1.09–1.30)].

**Conclusion:**

Persistent obesity and persistent abdominal obesity before the age of 50 are associated with a slightly increased risk of EO-CRC. Addressing obesity and abdominal obesity in young individuals might be beneficial in reducing the risk of EO-CRC.

## 1. Introduction

The incidence of early-onset colorectal cancer (EO-CRC) in individuals diagnosed with colorectal cancer (CRC) before 50 years of age has increased over the past three decades (1). EO-CRC now accounts for 10–12% of all newly diagnosed CRCs (2). Recently, the US Preventive Service Task Force expanded colorectal cancer screening recommendations to recommend screening for adults aged 45 years with and average risk profile (3). In a cohort study investigating the clinical characteristics of EO-CRC, it was found that the majority of patients exhibited symptoms such as rectal bleeding or abdominal pain (4). Additionally, left-sided cancer and advanced stages of the disease were commonly observed, and more than half of the patients were overweight or obese (4).

Obesity is known to be a risk factor for several major cancers, including CRC (5, 6). Recently, it was reported that not only overall obesity but also abdominal obesity are associated with an increased risk of colon cancer (CC) (7), cancer-related mortality (8), and all-cause mortality (8, 9).

Weight gain can increase the colorectal adenoma recurrence rate (10). A previous study reported that an increased waist circumference (WC) from early to later adulthood was associated with an increased risk of advanced colorectal neoplasia (11). Several studies have suggested that weight gain and an increased WC are associated with a higher risk of CRC (12, 13). The European Prospective Investigation into Cancer and Nutrition (EPIC) cohort study showed that losing weight and entering a lower body mass index (BMI) category in middle adulthood, between 40 and 70 years of age, lowered the risk of CC (5).

A previous study in Korea also demonstrated an association between EO-CRC risk and metabolic syndrome (MetS) and showed that a higher BMI and larger WC are important risk factors for EO-CRC (14).

We aimed to evaluate whether changes in BMI and WC are associated with EO-CRC risk and suggest how to manage them to prevent EO-CRC.

## 2. Materials and methods

### 2.1. Collection of data from the national health insurance corporation database

All Koreans join the National Health Insurance Service (NHIS), and universal medical coverage is provided in Korea. The data of all Koreans in the NHIS are managed by the National Health Insurance Corporation (NHIC). This patient database includes all kinds of medical information, such as demographic data, disease diagnoses, prescribed medicines, received treatments, and hospital visitation records. The NHIS also provides national health checkups every 2 years for all Koreans aged ≥40 years or any employees aged >20 years (15). Data from these health checkups include anthropometric data measured by licensed medical staff, lifestyle questionnaire data from self-surveys, blood and urine test results, and previous medical history. Since these data are obtained every 2 years, we could determine serial changes in one person. Therefore, the occurrence and diagnosis date of CRC were determined by reviewing the NHIC database.

### 2.2. Study population and flow

**Figure 1:**
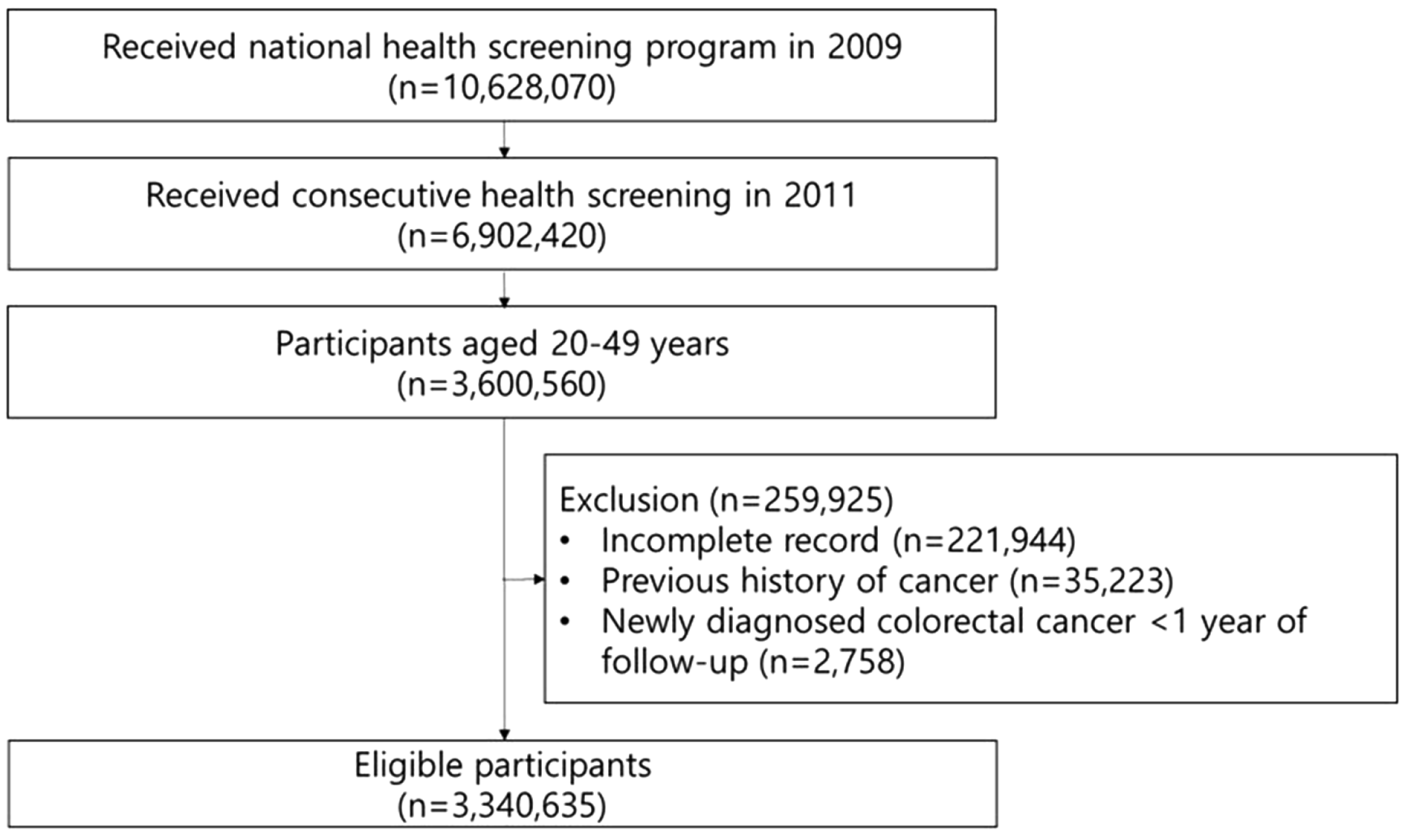
Flow chart of enrollment process in this study.

The flow of the enrollment process is displayed in Figure 1. Among the participants who participated in the national health screening program in both 2009 (S1) and 2011 (S2), those aged 20–49 years were included. Participants who had incomplete records (*n* = 221,944) or a previous history of cancer (*n* = 35,233) were excluded. Additionally, participants who were newly diagnosed with CRC or died from any cause within 1 year of follow-up (*n* = 2,758) were excluded, as the potential for preexisting CRC could not be considered. Ultimately, 3,340,635 participants were included in the study.

This study was approved by the Ethics Committee of Seoul National University Hospital (IRB No. E-2201-011-1,286) and conformed to the ethical guidelines of the World Medical Association’s Declaration of Helsinki. Since deidentified data were collected and used in this study, the requirement for informed consent from individual participants was waived.

### 2.3. Definitions of obesity, abdominal obesity and related variables

BMI was calculated as weight divided by height2 (kg/m2). According to the Asia-Pacific definition of the World Health Organization (WHO), a BMI ≥25 kg/m2 was defined as obesity, and a BMI <25 kg/m2 was defined as nonobesity (16). WC was measured by tape at the midpoint between the lower costal margin and anterior superior iliac crest (17). Abdominal obesity was defined as a WC ≥ 90 cm in men and ≥ 85 cm in women, according to the definition of the Korean Society for the Study of Obesity (18).

Lifestyle factors were recorded according to a self-survey. For smoking status, participants were considered to have never smoked or to have smoked in the past (no smoking group) or to currently smoke (smoking group). Alcohol intake was divided into no heavy drinking and heavy (average of ≥30 g of alcohol/day) drinking. Regular exercise was defined as performing moderate exercise ≥5 days per week or vigorous exercise ≥3 days per week. Low income was defined as an income in the lowest income quartile or receiving medical aid.

Hypertension was defined as a blood pressure ≥ 140/90 mmHg or a history of receiving antihypertensive medications. Diabetes was defined as a fasting glucose level ≥ 126 mg/dL or a history of receiving glucose-lowering agents. Dyslipidemia was defined as a total cholesterol level ≥ 240 mg/dL after a 12-h fast or a history of receiving dyslipidemia medication. MetS was defined when 3 or more of the following 5 criteria were satisfied: (1) a WC ≥90 cm for men or ≥ 85 cm for women; (2) a blood pressure ≥ 130/85 mm Hg or drug treatment for hypertension; (3) a fasting plasma glucose level ≥ 100 mg/dL or drug treatment for diabetes; (4) a serum triglyceride level ≥ 150 mg/dL or drug treatment for elevated triglycerides; and (5) a serum high-density lipoprotein cholesterol (HDL-C) level < 40 mg/dL in men or < 50 mg/dL in women.

### 2.4. Classification of changes in obesity and abdominal obesity status

Participants were classified by two methods according to obesity and abdominal obesity status, and follow-up results were analyzed separately. A change in obesity status was defined as a change in BMI measured at S1 and S2. Participants were divided into 4 groups: participants who did not have obesity at either examination (NNo), participants who did not have obesity at the first examination but had obesity at the second examination (NO), participants who had obesity at the first examination but did not have obesity at the second examination (ON), and participants who had obesity at both examinations (OO). A change in abdominal obesity status was defined as a change in WC measured at S1 and S2. Similar to participants with a change in obesity status, those with a change in abdominal obesity status were grouped into 4 categories: participants who did not have abdominal obesity at either examination (NNa), participants who did not have abdominal obesity at the first examination but had abdominal obesity at the second examination (NA), participants who had abdominal obesity at the first examination but did not have abdominal obesity at the second examination (AN), and participants who had abdominal obesity at both examinations (AA).

### 2.5. Study outcomes

The primary outcome of this study was new EO-CRC cases, which were diagnosed before 50 years of age. CRC was diagnosed according to the International Classification of Diseases Tenth Revision (ICD-10). The ICD-10 codes used in this study were as follows: C18-C20 for a CRC diagnosis, C18-19 for a CC diagnosis, C20.0 for a rectal cancer (RC) diagnosis, and the registration code for cancer [V193]. Registration of the cancer diagnosis and location was performed by a licensed doctor when the cancer was first diagnosed. Participants were followed until they were diagnosed with CRC or censored when they became older than 50 years.

### 2.6. Statistical analysis

Variables are expressed as the means ± standard deviations or numbers (percentages). To compare baseline characteristics among groups, ANOVA was used for continuous variables, and the χ2 test was applied for categorical variables. The incidence rates of CRC are presented as the number of events divided by 1,000 person-years. To evaluate EO-CRC risk, a multivariable Cox proportional hazard model was used. Participants were divided into 4 groups according to changes in obesity and abdominal obesity status. Hazard ratios (HRs) and 95% confidence intervals (CIs) were calculated without adjustment (Model 1), after adjustment for sex and age (Model 2), and after adjustment for sex, age, smoking status, alcohol consumption, regular exercise, income status, and MetS (Model 3). The correlation between the obesity group and abdominal obesity group was assessed using Cremér’s V. Sensitivity analyzes according to CRC subsites were performed. Finally, a Kaplan–Meier curve of EO-CRC was drawn after adjusting for sex, age, smoking status, alcohol consumption, regular exercise, income status, and MetS. Variables with *p* values <0.05 were considered statistically significant. Statistical analyzes were performed using SAS version 9.4 (SAS Institute Inc., Cary, NC, United States).

## 3. Results

### 3.1. Demographic and clinical characteristics

A total of 3,340,635 participants who received national health screenings in 2009 (S1) and 2011 (S2) were included in this study. During the mean 7.1-year follow-up period, 7,492 patients were diagnosed with CRC. The demographic and clinical characteristics of the group with changes in obesity and abdominal obesity status are shown in Table 1. The data presented in the tables were collected in S2.

**Table 1:** Baseline characteristics of the group with changes in obesity and abdominal obesity status.

	Total	Changes in obesity status					Changes in abdominal obesity status				
		NNo^a^	NO^a^	ON^a^	OO^a^	*P* value	NNa^b^	NA^b^	AN^b^	AA^b^	*P* value
	(*n* = 3,340,635)	(*n* = 2,157,875)	(*n* = 201,021)	(*n* = 128,953)	(*n* = 852,786)		(*n* = 2,666,690)	(*n* = 209,168)	(*n* = 153,928)	(*n* = 310,849)	
Sex (Male)	2,122,940 (63.6)	1,190,173 (55.2)	148,340 (73.8)	93,467 (72.5)	690,960 (81.0)	<0.001	1,592,076(59.7)	156,703 (74.9)	114,160 (74.2)	260,001(83.6)	<0.001
Age (yr)	38.3 ± 6.9	37.9 ± 7.1	38.0 ± 6.8	39.6 ± 6.5	39.2 ± 6.3	<0.001	38.1 ± 7.0	38.6 ± 6.6	39.8 ± 6.3	39.2 ± 6.3	<0.001
Current smoker	1,079,951 (32.3)	617,454 (28.6)	70,670 (35.2)	46,293 (35.9)	345,534 (40.5)	<0.001	807,122(30.3)	78,269 (37.4)	57,740 (37.5)	136,820(44.0)	<0.001
Heavy alcohol drinker^c^	289,752 (8.7)	146,979 (6.8)	21,385 (10.6)	12,651 (9.8)	108,737 (12.8)	<0.001	200,941(7.5)	25,472 (12.2)	17,644 (11.5)	45,695(14.7)	<0.001
Regular exercise^d^	583,556 (17.5)	351,818 (16.3)	34,248 (17.0)	30,992 (24.0)	166,498 (19.5)	<0.001	463,748(17.4)	32,663 (15.6)	32,969 (21.4)	54,176 (17.4)	<0.001
Low income^e^	424,479 (12.7)	291,682 (13.5)	23,172 (11.5)	16,606 (12.9)	93,019 (10.9)	<0.001	347,926(13.1)	23,722 (11.3)	19,536 (12.7)	33,295 (10.7)	<0.001
BMI (kg/m^2^)	23.6 ± 3.4	21.7 ± 2.0	25.8 ± 1.0	24.1 ± 0.9	27.8 ± 2.4	.	22.6 ± 2.6	26.8 ± 2.2	25.9 ± 2.2	29.2 ± 2.9	<0.001
WC (cm)	79.4 ± 9.4	75.0 ± 7.2	84.5 ± 5.7	81.0 ± 5.7	89.2 ± 7.2	<0.001	76.4 ± 7.3	91.5 ± 3.5	83.8 ± 4.9	95.5 ± 5.8	.
MetS	540,208 (16.2)	132,975 (6.2)	42,090 (20.9)	18,404 (14.3)	346,739 (40.7)	<0.001	208,723(7.8)	103,604 (49.5)	30,023 (19.5)	197,858 (63.7)	<0.001
Diabetes mellitus	130,071 (3.9)	50,098 (2.3)	7,135 (3.6)	7,892 (6.1)	64,946 (7.6)	<0.001	74,163(2.8)	11,415 (5.5)	11,193 (7.3)	33,300 (10.7)	<0.001
Hypertension	423,587 (12.7)	165,725 (7.7)	29,321 (14.6)	19,118 (14.9)	209,423 (24.6)	<0.001	252,135(9.5)	42,341 (20.2)	31,077 (20.2)	98,034 (31.5)	<0.001
Dyslipidemia	372,599 (11.2)	164,634 (7.6)	29,912 (14.9)	16,052 (12.5)	162,001 (19.0)	<0.001	240,131(9.0)	38,692 (18.5)	24,700 (16.1)	69,076 (22.2)	<0.001
Fasting glucose (mg/dL)	93.9 ± 19.0	91.7 ± 16.0	94.7 ± 16.3	96.6 ± 25.8	99.1 ± 23.6	<0.001	92.5 ± 16.8	96.8 ± 19.8	98.3 ± 25.7	102.1 ± 27.5	<0.001
Systolic BP (mm Hg)	119.6 ± 13.5	116.8 ± 12.8	122.6 ± 12.7	121.1 ± 12.8	125.9 ± 13.3	<0.001	118.1 ± 13.0	124.2 ± 13.2	123.4 ± 13.2	128.0 ± 13.6	<0.001
Diastolic BP (mm Hg)	75.4 ± 9.7	73.5 ± 9.2	77.2 ± 9.3	76.3 ± 9.3	79.6 ± 9.7	<0.001	74.3 ± 9.4	78.5 ± 9.72	77.8 ± 9.7	81.1 ± 10.0	<0.001
Total cholesterol (mg/dL)	191.7 ± 34.3	187.0 ± 32.7	199.5 ± 35.1	192.7 ± 34.3	201.9 ± 35.4	<0.001	189.2 ± 33.4	202.8 ± 36.0	197.1 ± 35.2	203.8 ± 36.1	<0.001
HDL-C (mg/dL)	55.8 ± 19.0	58.4 ± 19.3	52.7 ± 18.1	54.4 ± 18.7	50.2 ± 16.9	<0.001	57.2 ± 19.1	51.5 ± 18.0	52.0 ± 16.8	48.9 ± 16.8	<0.001
LDL-C (mg/dL)	110.7 ± 31.7	107.1 ± 30.1	116.6 ± 32.7	112.8 ± 31.8	118.1 ± 33.8	<0.001	108.9 ± 30.8	118.4 ± 33.5	115 ± 33.1	118.7 ± 34.8	<0.001
Triglycerides (mg/dL)	108.1 (108–108.1)	93.4 (93.3–93.5)	132.3 (132.0–132.6)	111.3 (111.0–111.7)	148.4 (148.2–148.6)	<0.001	99.6(99.6–99.7)	145.1 (144.8–145.4)	130.7 (130.4–131.1)	162.1 (161.8–162.5)	<0.001

Values are expressed as mean ± standard deviation, geometric mean (95% confidence interval) or frequencies (percentages).

AA, participants with abdominal obesity at both examinations; AN, participants with abdominal obesity at first examination who were normalized at second examination; BMI, body mass index; BP, blood pressure; HDL-C, high-density lipoprotein cholesterol; LDL-C, low-density lipoprotein cholesterol; MetS, metabolic syndrome; NA, participants without abdominal obesity at first examination but had abdominal obesity at second examination; NNa, participants without abdominal obesity at both examinations; NNo, participants who were not obese at both examinations; NO, participants who were not obese at first examination but became obese at second examination; ON, participants with obesity at first examination who became non-obese at second examination; OO, participants who were obese at both examinations; WC, waist circumference.

a Obesity is defined as BMI of ≥ 25 kg/m^2^.

b Abdominal obesity is defined as WC ≥ 90 cm in men and ≥ 85 cm in women.

c Heavy drinker is defined as average alcohol intake ≥ 30 g/d.

d Regular exercise is defined as moderate exercise ≥ 5 days or vigorous exercise ≥ 3 days in a week.

e Low income is defined as the lowest income quartile or receiving medical aid.

### 3.2. Association between changes in obesity or abdominal obesity status and EO-CRC risk

EO-CRC risk was stratified according to changes in obesity and abdominal obesity status (Table 2). Changes in obesity and abdominal obesity showed a moderate correlation (Cramér’s V = 0.39). After adjusting for sex, age, smoking status, alcohol consumption, regular exercise, income status, and MetS, EO-CRC risk was 9% higher in the OO group than in the NNo group. In the AA group, the increase in EO-CRC risk was even higher, up to 18%.

**Table 2:** Risk of earlier-onset colorectal cancer according to changes in obesity and abdominal obesity status.

	Obesity^a^	*N*	Events	Person-years	IR^c^	Model 1	Model 2	Model 3
Colorectal cancer	NNo	2,157,875	4,738	13,173,307	0.36	1 (Reference)	1 (Reference)	1 (Reference)
	NO	201,021	408	1,230,484	0.33	0.92 (0.83–1.02)	0.98 (0.89–1.09)	0.97 (0.88–1.08)
	ON	128,953	309	750,237	0.41	1.15 (1.02–1.29)	1.09 (0.97–1.23)	1.08 (0.96–1.21)
	OO	852,786	2,037	5,068,068	0.40	1.12 (1.06–1.18)	1.12 (1.06–1.18)	1.09 (1.03–1.16)
Colon cancer	NNo	2,157,875	3,495	13,173,307	0.27	1 (Reference)	1 (Reference)	1 (Reference)
	NO	201,021	280	1,230,484	0.23	0.86 (0.76–0.97)	0.96 (0.85–1.08)	0.95 (0.84–1.08)
	ON	128,953	217	750,237	0.29	1.09 (0.95–1.25)	1.09 (0.95–1.25)	1.08 (0.94–1.24)
	OO	852,786	1,400	5,068,068	0.28	1.04 (0.98–1.11)	1.12 (1.05–1.20)	1.10 (1.02–1.17)
Rectal cancer	NNo	2,157,875	1,243	13,173,307	0.09	1 (Reference)	1 (Reference)	1 (Reference)
	NO	201,021	128	1,230,484	0.10	1.10 (0.92–1.32)	1.03 (0.86–1.24)	1.02 (0.85–1.23)
	ON	128,953	92	750,237	0.12	1.30 (1.05–1.61)	1.10 (0.89–1.37)	1.09 (0.88–1.35)
	OO	852,786	637	5,068,068	0.13	1.33 (1.21–1.47)	1.12 (1.02–1.24)	1.09 (0.98–1.21)
	Abdominal obesity^b^	N	Events	Person-years	IR^c^	Model 1	Model 2	Model 3
Colorectal cancer	NNa	2,666,690	5,838	16,225,858	0.36	1 (Reference)	1 (Reference)	1 (Reference)
	NA	209,168	483	1,257,663	0.38	1.07 (0.97–1.17)	1.10 (1.00–1.21)	1.08 (0.98–1.19)
	AN	153,928	379	888,961	0.43	1.19 (1.07–1.32)	1.12 (1.01–1.24)	1.11 (1.00–1.23)
	AA	310,849	792	1,849,614	0.43	1.19 (1.11–1.28)	1.21 (1.12–1.31)	1.18 (1.09–1.29)
Colon cancer	NNa	2,666,690	4,248	16,225,858	0.26	1 (Reference)	1 (Reference)	1 (Reference)
	NA	209,168	337	1,257,663	0.27	1.02 (0.92–1.14)	1.10 (0.98–1.23)	1.08 (0.96–1.21)
	AN	153,928	276	888,961	0.31	1.19 (1.05–1.34)	1.16 (1.03–1.31)	1.15 (1.02–1.30)
	AA	310,849	531	1,849,614	0.29	1.10 (1.00–1.20)	1.20 (1.09–1.31)	1.16 (1.05–1.29)
Rectal cancer	NNa	2,666,690	1,590	16,225,858	0.10	1 (Reference)	1 (Reference)	1 (Reference)
	NA	209,168	146	1,257,663	0.12	1.19 (1.01–1.40)	1.10 (0.92–1.30)	1.08 (0.91–1.29)
	AN	153,928	103	888,961	0.12	1.18 (0.97–1.45)	1.02 (0.83–1.24)	1.01 (0.82–1.23)
	AA	310,849	261	1,849,614	0.14	1.44 (1.27–1.64)	1.24 (1.09–1.42)	1.22 (1.05–1.41)

AA, participants with abdominal obesity at both examinations; AN, participants with abdominal obesity at first examination who were normalized at second examination; IR, incidence rate; NA, participants without abdominal obesity at first examination but had abdominal obesity at second examination; NNa, participants without abdominal obesity at both examinations; NNo, participants without obesity at both examinations; NO, participants who were not obese at first examination but became obese at second examination; ON, participants with obesity at first examination who became non-obese at second examination; OO, participants who were obese at both examinations.

Model 1 was not adjusted.

Model 2 was adjusted for sex and age.

Model 3 was adjusted for sex, age, smoking status, alcohol consumption, regular exercise, low income status, and MetS.

a Obesity is defined as BMI of ≥ 25 kg/m^2^.

b Abdominal obesity is defined as WC ≥ 90 cm in men and ≥ 85 cm in women.

c Cancer incidence per 1,000 person-years.

**Figure 2:**
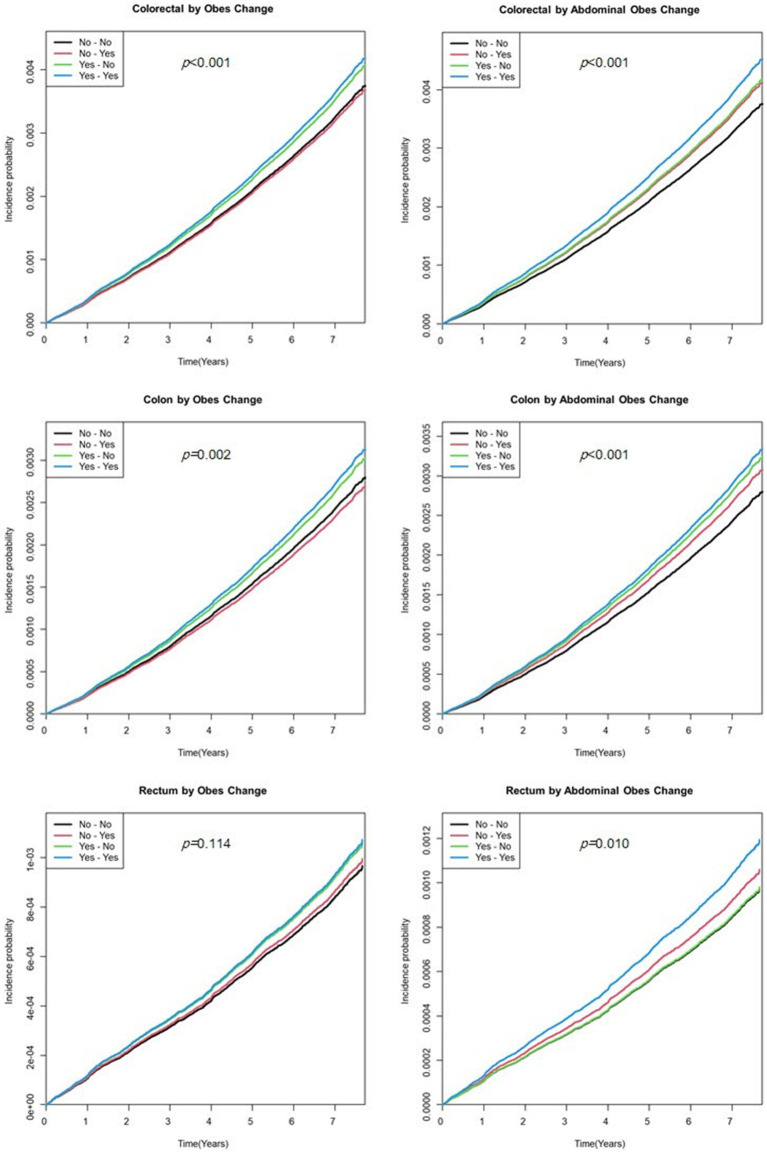
Kaplan–Meier curve of earlier-onset colorectal cancer by the cumulative obesity burden. Model was adjusted for sex, age, smoking status, alcohol consumption, regular exercise, and income status.

The cumulative incidence of EO-CRC according to obesity and abdominal obesity status is shown in Figure 2. Calculating EO-CRC risk according to the cumulative obesity burden (the number of participants diagnosed with obesity or abdominal obesity in S1 and S2), participants with cumulative obesity and abdominal obesity burdens of 2 had a higher incidence of EO-CRC compared with those with burdens of 0 (Figure 2). The incidence of EO-CRC, EO-CC, and EO-RC in participants with a cumulative abdominal obesity burden of 1 was drawn between 0 and 2.

### 3.3. Risk of EO-CRC classified according to two criteria: changes in obesity and abdominal obesity status

Participants were subdivided into 16 groups according to changes in obesity and abdominal obesity status (Table 3). Compared to the NNo-NNa group, the OO-AA group had a 19% higher EO-CRC risk. Interestingly, EO-CRC risk was significantly higher in participants whose BMIs became normal after being diagnosed with obesity but who newly developed abdominal obesity [ON NA, HR (95% CI) = 1.69 (1.04–2.77)] or persistent abdominal obesity [ON AA, HR (95% CI) = 1.87 (1.17–2.97)].

**Table 3:** The risk of earlier onset colorectal cancer when subdivided according to changes in obesity and abdominal obesity status.

Obesity^a^	Abdominal obesity^b^	*N*	Events	Person-years	IR^c^	Model1	Model2	Model3
NNo	NNa	2,091,451	4,569	12,796,159	0.36	1 (Reference)	1 (Reference)	1 (Reference)
NNo	NA	33,901	87	197,075	0.44	1.24 (1.00–1.53)	1.17 (0.95–1.45)	1.16 (0.94–1.44)
NNo	AN	26,490	68	148,502	0.46	1.29 (1.01–1.63)	1.14 (0.89–1.44)	1.13 (0.89–1.44)
NNo	AA	6,033	14	31,571	0.44	1.25 (0.74–2.11)	1.07 (0.64–1.82)	1.06 (0.62–1.79)
NO	NNa	151,991	296	932,513	0.32	0.89 (0.79–1.00)	0.94 (0.84–1.06)	0.94 (0.84–1.06)
NO	NA	36,643	84	227,526	0.37	1.03 (0.83–1.28)	1.15 (0.93–1.43)	1.14 (0.92–1.42)
NO	AN	5,677	13	32,107	0.40	1.14 (0.66–1.96)	1.05 (0.61–1.80)	1.04 (0.61–1.80)
NO	AA	6,710	15	38,339	0.39	1.10 (0.66–1.82)	1.07 (0.65–1.78)	1.05 (0.63–1.75)
ON	NNa	98,790	218	577,955	0.38	1.06 (0.92–1.21)	1.01 (0.88–1.16)	1.01 (0.88–1.15)
ON	NA	4,228	16	23,505	0.68	1.91 (1.17–3.13)	1.71 (1.05–2.80)	1.69 (1.04–2.77)
ON	AN	21,528	57	124,815	0.46	1.28 (0.99–1.66)	1.20 (0.93–1.56)	1.19 (0.92–1.55)
ON	AA	4,407	18	23,961	0.75	2.12 (1.33–3.36)	1.91 (1.20–3.03)	1.87 (1.17–2.97)
OO	NNa	324,458	755	1,919,231	0.39	1.10 (1.02–1.19)	1.08 (1.00–1.17)	1.08 (1.00–1.16)
OO	NA	134,396	296	809,557	0.37	1.03 (0.91–1.15)	1.06 (0.94–1.19)	1.04 (0.92–1.18)
OO	AN	100,233	241	583,537	0.41	1.16 (1.02–1.32)	1.11 (0.98–1.27)	1.10 (0.96–1.25)
OO	AA	293,699	745	1,755,743	0.42	1.19 (1.10–1.29)	1.22 (1.13–1.32)	1.19 (1.09–1.30)

A, abdominal obesity; N, participants without obesity or abdominal obesity; O, obesity.

NNo, participants who were not obese at both examinations; NO, participants who were not obese at first examination but became obese at second examination; ON, participants with obesity at first examination who became non-obese at second examination; OO, participants who were obese at both examinations; AA, participants with abdominal obesity at both examinations; AN, participants with abdominal obesity at first examination who were normalized at second examination; NA, participants without abdominal obesity at first examination but had abdominal obesity at second examination; NNa, participants without abdominal obesity at both examinations.

Model 1 was not adjusted.

Model 2 was adjusted for sex and age.

Model 3 was adjusted for sex, age, smoking status, alcohol consumption, regular exercise. Low income status, and MetS.

a Obesity is defined as BMI of ≥ 25 kg/m^2^.

b Abdominal obesity is defined as WC ≥ 90 cm in men and ≥ 85 cm in women.

c Cancer incidence per 1,000 person-years.

### 3.4. Sensitivity analysis

Sensitivity analyzes evaluating the risk factors for EO-CRC according to changes in obesity and abdominal obesity status were performed (Table 4). Males and females showed significantly different risks of EO-CC according to changes in obesity status (P for interaction = 0.005). Heavy drinkers in the AA group had a significantly higher risk of EO-CRC and EO-CC [HR (95% CI) = 1.30 (1.07–1.58) and 1.35 (1.06–1.72), respectively].

**Table 4:** Sensitivity analysis of risk of earlier-onset colorectal cancer according to changes in obesity and abdominal obesity status.

	Colorectal cancer, HR (95% CI)				Colon cancer, HR (95% CI)				Rectal cancer, HR (95% CI)			
	Obesity^a^ change				Obesity^a^ change				Obesity^a^ change			
	NNo	NO	ON	OO	NNo	NO	ON	OO	NNo	NO	ON	OO
Sex												
Male	1 (Ref)	1.01 (0.89,1.14)	1.10 (0.95,1.27)	1.18 (1.10,1.25)	1 (Ref)	1.05 (0.89,1.23)	1.16 (0.97,1.39)	1.23 (1.13,1.33)	1 (Ref)	0.94 (0.76,1.16)	1.01 (0.79,1.29)	1.09 (0.98,1.22)
Female	1 (Ref)	0.96 (0.81,1.14)	1.08 (0.89,1.31)	1.00 (0.90,1.10)	1 (Ref)	0.87 (0.72,1.06)	1.01 (0.81,1.25)	0.97 (0.87,1.08)	1 (Ref)	1.40 (0.98,1.99)	1.44 (0.95,2.19)	1.14 (0.90,1.44)
*p* for interaction			0.058				0.005				0.147	
Age group												
20s	1 (Ref)	1.04 (0.68,1.59)	1.92 (1.20,3.09)	1.29 (1.00,1.68)	1(Ref.)	1.03 (0.61,1.74)	1.71 (0.93,3.13)	1.30 (0.94,1.79)	1(Ref.)	1.00 (0.48,2.06)	2.31 (1.07,4.99)	1.20 (0.77,1.89)
30s	1 (Ref)	1.00 (0.85,1.19)	1.18 (0.96,1.44)	1.11 (1.01,1.21)	1 (Ref)	0.97 (0.78,1.20)	1.22 (0.96,1.6)	1.08 (0.96,1.20)	1 (Ref)	1.06 (0.80,1.41)	1.08 (0.76,1.55)	1.16 (0.99,1.35)
40s	1 (Ref)	0.97 (0.85,1.10)	1.00 (0.86,1.16)	1.11 (1.04,1.19)	1 (Ref)	0.95 (0.81,1.11)	0.99 (0.83,1.18)	1.13 (1.05,1.22)	1 (Ref)	1.01 (0.79,1.30)	1.03 (0.78,1.36)	1.07 (0.94,1.22)
*p* for interaction			0.217				0.458				0.619	
Smoking status												
Non	1 (Ref)	1.01 (0.90,1.14)	1.12 (0.97,1.28)	1.08 (1.01,1.16)	1 (Ref)	0.97 (0.84,1.12)	1.04 (0.88,1.23)	1.08 (1.00,1.17)	1 (Ref)	1.12 (0.89,1.40)	1.33 (1.03,1.71)	1.08 (0.94,1.22)
Current	1 (Ref)	0.92 (0.76,1.11)	1.03 (0.84,1.26)	1.18 (1.08,1.29)	1 (Ref)	0.93 (0.74,1.18)	1.18 (0.93,1.51)	1.20 (1.07,1.34)	1 (Ref)	0.89 (0.65,1.22)	0.76 (0.51,1.13)	1.15 (0.99,1.33)
*p* for interaction			0.227				0.414				0.048	
Alcohol consumption												
Non	1 (Ref)	0.95 (0.85,1.06)	1.11 (0.99,1.26)	1.11 (1.05,1.17)	1 (Ref)	0.94 (0.83,1.07)	1.10 (0.95,1.27)	1.10 (1.03,1.18)	1 (Ref)	0.97 (0.80,1.19)	1.15 (0.92,1.44)	1.12 (1.00,1.24)
Heavy drinker^c^	1 (Ref)	1.27 (0.95,1.69)	0.83 (0.54,1.27)	1.20(1.02,1.41)	1 (Ref)	1.18 (0.80,1.74)	0.90 (0.53,1.53)	1.28 (1.04,1.57)	1 (Ref)	1.38 (0.89,2.14)	0.72 (0.35,1.48)	1.08 (0.83,1.41)
*p* for interaction			0.108				0.335				0.254	
Physical activity												
Non	1 (Ref)	0.97 (0.87,1.09)	1.12 (0.98,1.27)	1.13 (1.06,1.20)	1 (Ref)	0.93 (0.81,1.07)	1.10 (0.94,1.29)	1.12 (1.05,1.21)	1 (Ref)	1.05 (0.86,1.29)	1.15 (0.90,1.46)	1.13 (1.01,1.26)
Regular exercise^d^	1 (Ref)	1.04 (0.83,1.31)	0.10 (0.78,1.26)	1.08 (0.96,1.22)	1 (Ref)	1.09 (0.83,1.43)	1.02 (0.77,1.35)	1.11 (0.96,1.27)	1 (Ref)	0.93 (0.60,1.43)	0.94 (0.60,1.47)	1.03 (0.83,1.28)
*p* for interaction			0.713				0.705				0.768	
Economic status												
Other than low	1 (Ref)	0.98 (0.88,1.09)	1.06 (0.94,1.21)	1.12 (1.06,1.19)	1 (Ref)	0.95 (0.83,1.09)	1.07 (0.92,1.24)	1.14 (1.06,1.22)	1 (Ref)	1.04 (0.86,1.26)	1.05 (0.84,1.32)	1.10 (0.99,1.22)
Low income^e^	1 (Ref)	0.99 (0.75,1.31)	1.23 (0.92,1.64)	1.07 (0.92,1.24)	1 (Ref)	1.01 (0.74,1.39)	1.15 (0.82,1.62)	1.02 (0.86,1.21)	1 (Ref)	0.92 (0.50,1.71)	1.50 (0.85,2.64)	1.22 (0.90,1.64)
*p* for interaction			0.696				0.604				0.620	

	Colorectal cancer, HR (95% CI)				Colon cancer, HR (95% CI)				Rectal cancer, HR (95% CI)			
	Abdominal obesity^b^ change				Abdominal obesity^b^ change				Abdominal obesity^b^ change			
	NNa	NA	AN	AA	NNa	NA	AN	AA	NNa	NA	AN	AA
Sex												
Male	1 (Ref)	1.11 (0.99,1.25)	1.15 (1.01,1.30)	1.24 (1.14,1.35)	1 (Ref)	1.15 (1.00,1.32)	1.23 (1.06,1.44)	1.26 (1.13,1.41)		1.06 (0.87,1.28)	1.00 (0.80,1.25)	1.21(1.05,1.39)
Female	1 (Ref)	1.07 (0.91,1.26)	1.05 (0.87,1.26)	1.09 (0.93,1.28)	1 (Ref)	1.04 (0.87,1.24)	1.05 (0.86,1.29)	1.04 (0.87,1.25)		1.22 (0.84,1.77)	1.02 (0.65,1.62)	1.34 (0.94,1.92)
*p* for interaction			0.493				0.180				0.881	
Age group												
20s	1 (Ref)	0.92 (0.57,1.47)	1.14 (0.62,2.07)	1.33 (0.89,1.98)	1 (Ref)	0.68 (0.35,1.32)	1.35 (0.70,2.63)	1.02 (0.58,1.77)	1 (Ref)	1.37 (0.69,2.70)	0.65 (0.16,2.63)	1.84 (1.03,3.27)
30s	1 (Ref)	1.03 (0.88,1.21)	1.15 (0.96,1.38)	1.18 (1.04,1.34)	1 (Ref)	1.08 (0.89,1.31)	1.16 (0.92,1.44)	1.08 (0.91,1.27)	1 (Ref)	0.92 (0.69,1.24)	1.13 (0.82,1.55)	1.34 (1.10,1.64)
40s	1 (Ref)	1.15 (1.02,1.29)	1.09 (0.96,1.24)	1.21 (1.10,1.33)	1 (Ref)	1.14 (0.99,1.31)	1.15 (0.99,1.34)	1.26 (1.13,1.41)	1 (Ref)	1.18 (0.95,1.47)	0.95 (0.73,1.24)	1.11 (0.92,1.33)
*p* for interaction			0.878				0.512				0.286	
Smoking status												
Non	1 (Ref)	1.07 (0.95,1.20)	1.09 (0.96,1.24)	1.16 (1.05,1.28)	1 (Ref)	1.06 (0.93,1.22)	1.13 (0.98,1.32)	1.16 (1.03,1.30)	1 (Ref)	1.08 (0.87,1.35)	0.97 (0.74,1.26)	1.15 (0.96,1.39)
Current	1 (Ref)	1.16 (0.99,1.35)	1.16 (0.97,1.38)	1.28 (1.14,1.43)	1 (Ref)	1.19 (0.98,1.44)	1.21 (0.98,1.49)	1.26 (1.09,1.45)	1 (Ref)	1.10 (0.85,1.44)	1.07 (0.79,1.45)	1.32 (1.09,1.59)
*p* for interaction			0.529				0.669				0.773	
Alcohol consumption												
Non	1 (Ref)	1.16 (1.05,1.28)	1.10 (0.98,1.22)	1.18 (1.09,1.29)	1 (Ref)	1.15 (1.03,1.29)	1.15 (1.01,1.31)	1.17 (1.06,1.29)	1 (Ref)	1.16 (0.97,1.39)	0.96 (0.77,1.20)	1.22 (1.06,1.42)
Heavy drinker^c^	1 (Ref)	0.67 (0.48,0.93)	1.22 (0.91,1.63)	1.30 (1.07,1.58)	1 (Ref)	0.65 (0.42,1.01)	1.20 (0.82,1.75)	1.35(1.06,1.72)	1 (Ref)	0.69(0.41,1.17)	1.26 (0.79,2.00)	1.23 (0.89,1.68)
*p* for interaction			0.008				0.041				0.181	
Physical activity												
Non	1 (Ref)	1.12 (1.01,1.24)	1.17 (1.04,1.31)	1.19 (1.10,1.30)	1 (Ref)	1.13 (1.00,1.27)	1.20 (1.05,1.37)	1.18 (1.07,1.31)	1 (Ref)	1.11 (0.92,1.33)	1.10 (0.88,1.36)	1.21 (1.04,1.40)
Regular exercise^d^	1 (Ref)	0.99 (0.78,1.25)	0.92(0.72,1.16)	1.25 (1.06,1.48)	1 (Ref)	0.98 (0.74,1.30)	1.01 (0.77,1.32)	1.23 (1.00,1.51)	1 (Ref)	1.01 (0.66,1.54)	0.71 (0.44,1.16)	1.30 (0.97,1.74)
*p* for interaction			0.212				0.539				0.397	
Economic status												
Other than low	1 (Ref)	1.10 (1.00,1.22)	1.14 (1.02,1.27)	1.20 (1.11,1.31)	1 (Ref)	1.09 (0.97,1.23)	1.17 (1.02,1.33)	1.19 (1.08,1.32)	1 (Ref)	1.12 (0.94,1.34)	1.07 (0.87,1.31)	1.23 (1.07,1.41)
Low income^e^	1 (Ref)	1.07 (0.82,1.39)	0.95 (0.70,1.28)	1.2 (0.97,1.49)	1 (Ref)	1.15 (0.86,1.54)	1.08 (0.78,1.50)	1.18 (0.91,1.52)	1 (Ref)	0.82 (0.45,1.50)	0.53 (0.23,1.19)	1.25 (0.82,1.89)
*p* for interaction			0.746				0.960				0.308	

HR, hazard ratio; CI, confidence interval; NNo, participants who were not obese at both examinations; NO, participants who were not obese at first examination but became obese at second examination; ON, participants with obesity at first examination who became non-obese at second examination; OO, participants who were obese at both examinations.

Hazard ratio (95% CI) was adjusted for sex, age, smoking status, alcohol consumption, physical activity, and economic status.

a Obesity is defined as BMI of ≥ 25 kg/m^2^.

b Abdominal obesity is defined as WC ≥ 90 cm in men and ≥ 85 cm in women.

c Heavy drinker is defined as average alcohol intake ≥ 30 g/d.

d Regular exercise is defined as moderate exercise ≥ 5 days or vigorous exercise ≥ 3 days in a week.

e Low income is defined as the lowest income quartile or receiving medical aid.

## 4. Discussion

According to this large nationwide population-based cohort study, the incidence of EO-CRC showed a slight increase in individuals with persistent obesity (9%) or persistent abdominal obesity (18%). Having both persistent obesity and abdominal obesity further increased EO-CRC risk (19%).

To our knowledge, this is the first study to clarify the association between changes in obesity status and EO-CRC risk. We also compared the effect of changes in obesity and abdominal obesity status. A prospective cohort study evaluating CRC risk according to the degree of weight change revealed that an increased WC and weight during middle adult life were not associated with CRC risk (19). Another prospective cohort study showed that abdominal adiposity was associated with an increased CRC risk in men (13). These studies differ from our study in that the ages of the subjects ranged from 40 to 69 years, or the average participant was in their 60s.

Referring to the baseline characteristics, the percentages of NNo, NO, ON, and OO were 64.6, 6.0, 3.9, and 25.5%, respectively, for changes in obesity status in this study. Although it was a short time interval, the groups changed for only a small number of participants. This means that lean people remain lean and obese people remain obese. A previous meta-analysis showed that obesity persisted from childhood to adulthood (20). Childhood obesity may affect CRC risk later in life (21, 22). The importance of childhood obesity should be emphasized and interventions should be provided.

Interestingly, it was observed that even in cases of weight loss, the risk of EO-CRC was higher when abdominal obesity newly developed (ON NA, HR 1.69) or persisted (ON AA, HR 1.87). These findings suggest that abdominal obesity may have a greater influence on the development of EO-CRC compared to obesity measured by BMI. There have been several studies consistent with these results. A study reported that abdominal fat, independent of overall obesity, was associated with an increased CRC risk in men (13). Another study suggested that a WC change might be a better predictor of advanced colorectal neoplasia than a BMI change (11). Recently, another study reported that a large WC was associated with colorectal neoplasia risk even with a normal weight (23). The plausible mechanisms by which abdominal obesity increases CRC risk include insulin resistance, hyperinsulinemia, chronic inflammation, and altered levels of growth factors, adipocytokines and steroid hormones (24). However, it should be noted that the number of participants in these groups (ON NA, ON AA) was relatively small, which limits the certainty of these conclusions. Further studies with a larger sample size are needed to provide more conclusive evidence.

In the sensitivity analyzes to evaluate the risk factors for EO-CRC according to changes in obesity status, persistent obesity increased EO-CC risk in men but not women. These results were similar to those of previous studies on CRC (25). In the presence of persistent abdominal obesity, participants who reported heavy drinking had a 30% higher risk of developing EO-CRC. A previous meta-analysis of risk factors for EO-CRC showed that high levels of alcohol consumption increased EO-CRC risk (26). However, there has been no report on the effect of alcohol consumption on EO-CRC risk according to changes in abdominal obesity status.

There are several limitations in this study. First, this was a retrospective study, and the follow-up period was relatively short because of the age restriction of the outcome. Second, the diagnostic criteria of obesity (16) and abdominal obesity (18) have been defined for Asians and are different from the WHO criteria. Last, the gap between S1 and S2 was too short to reflect long-term changes in obesity status. To evaluate EO-CRC risk in normal-to-obese and obese-to-normal groups, it is necessary to follow up changes over a period of 2 years or more. Intervals greater than 2 years were not investigated in this study due to insufficient data. Further studies are needed to determine the long-term effects of changes in obesity and abdominal obesity status.

Nevertheless, this study is significant in that changes in obesity and abdominal obesity status were analyzed, which are modifiable risk factors among EO-CRC patients in a large population. Although CRC screening guidelines recommend starting colonoscopy at the age of 50 years, initial colonoscopy at an earlier age should be recommended for younger people who have persistent obesity or are lean but have abdominal obesity. To overcome the limitations of this study, we plan to study how long-term changes in obesity and abdominal obesity status over more than 5 years affect EO-CRC patients.

In conclusion, individuals below the age of 50 who have persistent obesity or persistent abdominal obesity are at a slightly higher risk of developing EO-CRC compared to non-obese individuals. Moreover, the risk of EO-CRC increases with the number of cumulative diagnoses of obesity and abdominal obesity. Therefore, monitoring and intervening to address obesity and abdominal obesity in young individuals may be beneficial in reducing the risk of EO-CRC.

## Data availability statement

The raw data supporting the conclusions of this article will be made available by the authors, without undue reservation.

## Ethics statement

The studies involving human participants were reviewed and approved by the Ethics Committee of Seoul National University Hospital (IRB No. E-2201-011-1286). Written informed consent for participation was not required for this study in accordance with the national legislation and the institutional requirements.

## Author contributions

JHS and JYS: conceptualization, interpretation of data, and writing–original draft. EHJ, GEC, YSK, JHB, and SK: interpretation of data. K-DH: conceptualization, formal analysis, interpretation of data, methodology, resources, software, and supervision. SYY: conceptualization, interpretation of data, supervision, and writing–review and editing. All authors contributed to manuscript revision, read, and approved the submitted version.

## Conflict of interest

The authors declare that the research was conducted in the absence of any commercial or financial relationships that could be construed as a potential conflict of interest.

## Publisher’s note

All claims expressed in this article are solely those of the authors and do not necessarily represent those of their affiliated organizations, or those of the publisher, the editors and the reviewers. Any product that may be evaluated in this article, or claim that may be made by its manufacturer, is not guaranteed or endorsed by the publisher.

## Abbreviations

BMI: body mass index
CC: colon cancer
CI: confidence interval
CRC: colorectal cancer
EO: early onset
HbA1C: glycated hemoglobin level
HDL-C: high-density lipoprotein cholesterol
HR: hazard ratio
ICD-10: International Classification of Diseases Tenth Revision
MetS: metabolic syndrome
NHIC: National Health Insurance Corporation
NHIS: National Health Insurance Service
RC: rectal cancer
S1: national health screening performed in 2009
S2: national health screening performed in 2011
WC: waist circumference
WHO: World Health Organization
NNa: participants who did not have abdominal obesity (WC <90 cm in men and < 85 cm in women) at either examinations
NA: participants who did not have abdominal obesity at the first examination but had abdominal obesity at the second examination
AN: participants who had abdominal obesity at the first examination but did not have abdominal obesity at the second examination
AA: participants who had abdominal obesity at both examinations
NNo: participants who did not have obesity (BMI < 25 kg/m^2^) at either examinations
NO: participants who did not have obesity at the first examination but had obesity at the second examination
ON: participants who had obesity at the first examination but did not have obesity at the second examination
OO: participants who had obesity at both examinations
